# Acellular Dermal Matrices and Radiotherapy in Breast Reconstruction: A Systematic Review and Meta-Analysis of the Literature

**DOI:** 10.1155/2014/472604

**Published:** 2014-05-21

**Authors:** Luigi Valdatta, Anna Giulia Cattaneo, Igor Pellegatta, Stefano Scamoni, Anna Minuti, Mario Cherubino

**Affiliations:** ^1^Department of Biotechnology & Life Science (DBSV), University of Insubria, 21100 Varese, Italy; ^2^Plastic and Reconstructive Surgery Division, Ospedale di Circolo di Varese, Viale Borri 57, 21100 Varese, Italy

## Abstract

The increasing use of commercially available acellular dermis matrices for postmastectomy breast reconstruction seems to have simplified the surgical procedure and enhanced the outcome. These materials, generally considered to be highly safe or with only minor contraindications due to the necessary manipulation in preparatory phases, allow an easier one-phase surgical procedure, in comparison with autologous flaps, offering a high patient satisfaction. Unfortunately, the claim for a higher rate of complications associated with irradiation at the implant site, especially when the radiation therapy was given before the reconstructive surgery, suggested a careful behaviour when this technique is preferred. However, this hypothesis was never submitted to a crucial test, and data supporting it are often discordant or incomplete. To provide a comprehensive analysis of the field, we searched and systematically reviewed papers published after year 2005 and registered clinical trials. On the basis of a meta-analysis of data, we conclude that the negative effect of the radiotherapy on the breast reconstruction seems to be evident even in the case of acellular dermis matrices aided surgery. However, more trials are needed to make solid conclusions and clarify the poor comprehension of all the factors negatively influencing outcome.

## 1. Introduction

The acellular dermis matrices (ADM) are products derived from the skin, deprived of their cellular component by standardized treatments [1]. They provide a lower-lateral coverage and support of the implants in the immediate expander/implant-based breast reconstruction after mastectomy. Additional main indications for their use are lack of muscular coverage and cancer invasion to the pectoralis major muscle, and skin nipple sparing mastectomy is a relative indication [2].

The customized commercial products mainly differ in their origin and in procedures for processing, storing, and preparing them before usage. A recent paper compared seven customized ADM suitable for the reconstruction of the breast, in order to evaluate their cost/benefit ratios, contraindications, and possible side effects [3]. These authors discuss main contraindications for the use of commercial products, among others, namely, the presence of residues of antibiotics or allergenic substances, the lack of sterility, lower strength and elasticity, and higher cost. The Alloderm (LifeCell Corporation, Branchburg, NJ, USA), a customized derivative of the banked human skin, is the most widely used material, despite a few disadvantages: longer rehydration time, possible presence of antibiotics, and nonsterility of the final product.

We analyzed the data reported in peer-reviewed papers, in which irradiation at the site of ADM implant and its timing are considered as possible interfering factors for surgical outcome. The review includes an exhaustive description of the procedure followed for recording and selecting the published data, followed by their analysis and comment.

## 2. Materials and Methods

### 2.1. Search Strategy: Inclusion and Exclusion Criteria

The electronic search was coordinated, according to the Boolean syntax, in the following format: ((“acellular dermis” OR “acellular dermal matrix” OR Alloderm OR Strattice OR allomax OR Permacol OR Surgimed) AND (“breast reconstruction” OR mammoplasty OR mammaplasty)). The names of two commercial products (Flex-HD and DermaMatrix) generated ambiguity and therefore were omitted. The additional terms ((radiation) OR irradiation) OR RXT, coordinated with the previous terms by AND, restricted the search to the main aim of this review. A manual refinement rejected duplicate studies (those present in multiple databases), those performed “in vitro” or in animals, and studies not reporting original results or in which radiotherapy (RXT) was not directly investigated. Only the most recently updated studies were used. The reviews were carefully read, and other original reports eventually not found by the electronic procedure were retrieved and considered. The inclusion and exclusion criteria are reported in Table 1.

### 2.2. Databases

The bibliographic search was performed (final updating: 21st February, 2014) at the PubMed (US National Library of Medicine; http://www.ncbi.nlm.nih.gov/ pubmed) and at the Cochrane library (http://www.thecochranelibrary.com/view/0/index.html). The completeness of results was finally checked with a web-based tool, provided by the library of our institution and searching in different databases (Science Citation Index, Medline, Springer Link, Walters Kluwer, Ovid, and Cross Ref).

We also considered the studies registered at the clinical trials (USA, http://www.clinicaltrials.gov/) and at the international clinical trials registry platform (World Health Organisation, http://apps.who.int/trialsearch/Default.aspx) (Table 2).

### 2.3. Data Collection and Analysis

Data collection included the lead author, publication year, type of acellular dermal matrix, time range of study, total number of patients, and total number of reconstructions, with and without irradiation. The total number and type of complications in each group were recorded, as was the timing of RXT, before or after the reconstruction. Chemotherapy was also considered, when specified. The authors carefully followed the guidelines of the meta-analysis of observational studies in epidemiology [5].

### 2.4. Statistics

We extracted data from the papers which matched the inclusion criteria to evaluate the excessive risk for complications due to the adjuvant RXT given at the site of breast reconstruction. The forest and funnel plots of the odds ratios with confidence intervals at 95% and log odds ratios versus the standard error were built; the test of null and heterogeneity were calculated under a fixed model. For the necessary calculation we used the comprehensive meta-analysis software version 2.2.064 (released July 27, 2011) [6].

## 3. Results

### 3.1. Selected Literature and Main Features

**Figure 1:**
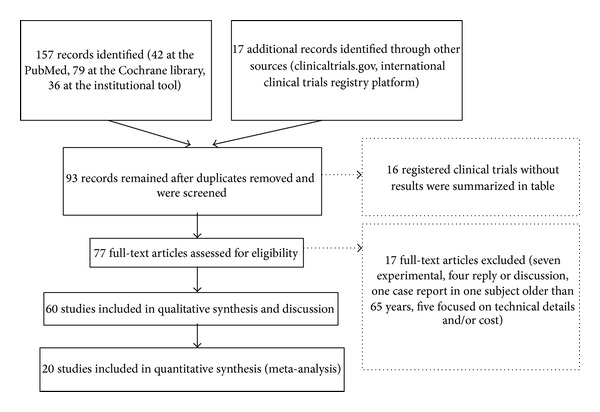
The strategy followed for the selection of the literature. All papers were carefully matched with the inclusion/exclusion criteria. Three search engines were used, for a total of 8 independent databases, as explained in the algorithm.

The algorithm for the selection of the literature matching the inclusion criteria is explained in Figure 1. The limited number of studies retrieved at the PubMed (*n* = 234 before the terms “radiation,” “radiotherapy,” and “RXT” were included) represented no more than 0.04% of all the papers describing different methods of breast reconstruction, in relation with RXT, and present in the same database under the period 2005–2014. This number was reduced to 51 when the terms ((radiation) OR irradiation) OR RXT were added. The search at the Cochrane library and at the institutional web-based tools gave 79 and 36 additional results, respectively. The 97 items remaining after manual revision for duplicates included 29 papers lacking extractable data but with relevant considerations or findings and 17 registered clinical trials (Table 2), one of which with published results [4]. Twenty works added results and were included in the meta-analysis (Table 3). Thirty-two papers (reviews and other publications without extractable data) were excluded.

The level of evidence, according to the rating scale issued by the American Society of Plastic Surgeons [27], was low; 80% of the selected papers scored 3 and the remaining 20% scored 4.

**Figure 2:**
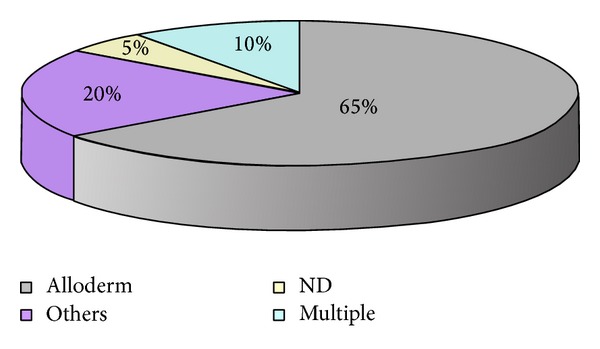
The use of different types of acellular dermis matrices in selected studies. Only the 60 papers included in the review were considered. The Alloderm (grey) was used in 65%, 20% used other acellular dermis matrices (fuchsia), 10% multiple ADM (pale blue), and 5% of authors did not specify the type of ADM they used (yellow).

The Alloderm is the most exploited and studied type of ADM (Figure 2); studies on other commercially available products are sporadic, frequently aimed to compare different materials and procedures.

### 3.2. Meta-Analysis

**Figure 3:**
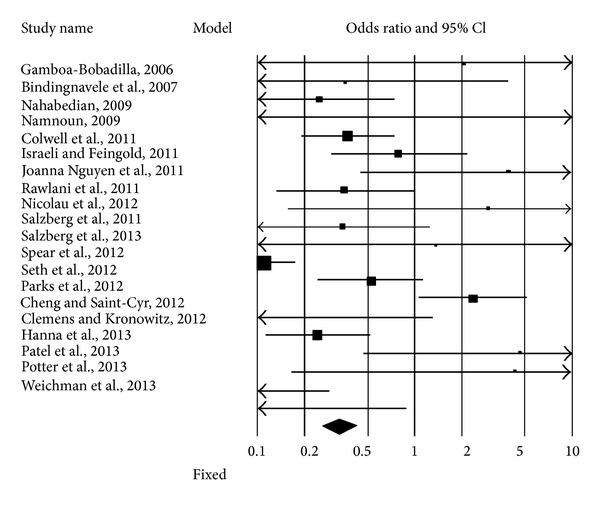
Forest plot of 20 studies. The authors reported the complications occurring in ADM-assisted immediate implant breast reconstruction, with or without radiotherapy (RXT). Odds ratios and confidence intervals at 95% are plotted. The black diamond at the bottom is the pooled odds ratio and its CI 95%. It completely falls to the left of 1.0 (<1), meaning that RXT significantly increases the risk of complications.

**Figure 4:**
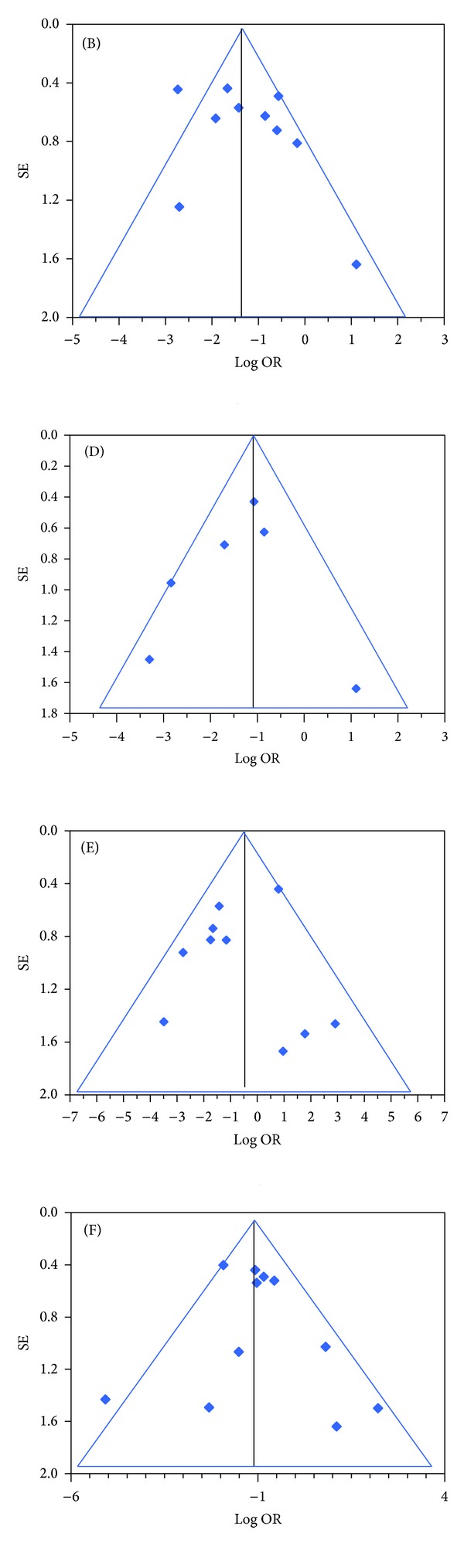
Funnel plots of 20 studies. Complications in ADM-assisted immediate implant breast reconstruction with or without RXT, occurring with statistically significant difference. (B) Skin necrosis; (D) capsular contracture; (E) other complications, sparsely described; (F) failure.

We extracted the data for statistical analysis from twenty studies matching our inclusion criteria, which reported the outcome of 3331 ADM-assisted reconstructions. They were mainly retrospective cohort studies, in which the observation period ranged between the years 2001 and 2012. With the exception of two works, performed in UK [8] and Canada [7], one study reported the results obtained in a private practice in USA [28] and the others were done in departments of hospitals and universities in the USA. Sixteen percent of reconstructions were irradiated before, after, or before and after surgery; in these cases the rate of development for complications was 33%. The percentage of nonirradiated breasts which developed at least one complication was instead lower (6%). The forest plot (Figure 3) shows the odds ratios of each study; the black diamond at the bottom represents the pooled effect. It fell on the left of level 1 and does not cross it; this result means that a significantly higher number of complications occurred when the ADM-assisted breast reconstruction was combined with RXT. In particular, eight studies found better results in the absence of RXT and only one [21] disagreed. The test of null was significant in both cases (*Z* value = −8.841, *P* value < 0.000); the complete results are shown in Tables 4 and 5. The funnel plots (Figure 4) show an acceptable distribution of the log odds ratio.

No more than 11 studies clearly specified the prevalence of implant failure and the most frequent type of complications in the two groups with or without RXT, as shown in Table 6, with higher prevalence of skin necrosis and infection. The prevalence of capsular contracture, reported in seven studies, was low (3%) but increased to 12% in irradiated reconstructions. Others conditions, such as wound dehiscence, haematoma, rippling, or implant migration, were not homogenously reported by all authors; therefore, we have grouped them. The RXT significantly enhanced the risk for skin necrosis and capsular contracture, as well as the implant failure due to different conditions.

## 4. Discussion

The RXT as adjuvant therapy for patients affected by mammary cancer is a trouble for the plastic surgeons as it could cause a tissue insult possibly affecting the final outcome of postmastectomy breast reconstruction. The relatively recent introduction of ADM-assisted implant reconstruction seemed to help a better procedure; however, its safety when RXT is needed is still under debate and experimental data are scanty and not conclusive. In many cases, as in the majority of clinical trials more recently registered at the USA repository, a history of previous irradiation is sufficient to exclude the use of the ADM and prefer an autologous reconstruction instead. In general, the authors of meta-analyses and traditional reviews agreed that the RXT enhances the risk of complications; however, no general agreement was reached, nor all works were conclusive in this aspect. A recent survey evaluated that at least 25% of patients submitted to postmastectomy breast reconstruction, independently from the use of ADM, and received prereconstruction RXT. The prevalence was large, exceeding 50%, in those which received postmastectomy RXT [29]. The relatively low rate of RXT (16% of all reconstructions considered) in this work could eventually reflect a bias in the choice of the technique, entailing that the autologous reconstruction could be preferred to the use of the ADM when the RXT was performed before surgery or expected to be needed later. While the first case could be confirmed at least in part by the general attitude of considering previous irradiation as an exclusion criteria for the use of expensive ADM (see Table 2), the second remains largely hypothetical.

The rational of these warnings resides in the nature of the ADM. They are generally protein derivatives, mainly composed of collagen, to which elastin, proteoglycans, and glycosaminoglycans may be added. The impact with ionizing radiation to sterilize the dried form of Alloderm affected the 3D structure of the collagen matrix, and different species of free radicals developed [28, 30, 31]. An unsolved issue is how the modalities of irradiation could be modified to reduce the impact on the skin and on the implant. In addition to the timing, which remains a possible additional risk factor (that was, however, not supported by the experimental evidence), the hypofractionation of the total dose of radiation (40 Gy in 15 fractions over 3 weeks) was associated with a higher incidence of severe capsular contracture. A clinical study with similar aim describes the changes of the native capsule architecture in ADM- and non-ADM-assisted reconstructions, before and after RXT [32]. In the presence of ADM and before RXT, the amount of elastin fibers was roughly duplicated and the number of macrophages fivefold reduced. However, when the ADM was used, irradiation did not induce relevant changes in the native capsule, not even excessive neovascularisation. The authors used Alloderm, Strattice, and NeoForm. This work did not report the modalities of irradiation, not even the incidence of complications in no ADM-assisted reconstructions. In 27 reconstructions with ADM, nine capsular contracture (grade III-IV) and nine different complications developed after irradiation. Nine complications exited in implant failure. No capsular contraction nor failure, and only three complications developed in the absence of irradiation.

The results of the meta-analysis presented in this review, in which three previously not reported results have been included [7, 8, 26], supported the thesis that the RXT represents a serious challenge; the influence of its timing in relation with surgery is, however, not noticeable due to the scarcity of data. The general opinion that RXT is a risk for higher rate of complications is, however, a controversial issue. Only few authors of original works lacking extractable data unequivocally concluded that RXT enhanced risk of complications [33–38]. Unfortunately, the only study in the field classified at level 1 of evidence only named RXT among the exclusion criteria [39]. Ibrahim et al., 2013 [40], recently found in a large database (19100 patients identified) the only association of greater risk for postoperative urinary tract infection in the group receiving the ADM and RXT. The authors admitted that unconventional results could be due to a bias derived from the very large number of cases in their analysis. To this, it could be added as an emerging opinion that in the context of greater morbidity due to the additional insult of RXT, the use of ADM seems to be protective and reduces the rate of complications, in particular capsular contracture [41–43]. While a large number of researchers prefer to behave cautiously, as documented by the strategy adopted by the majority of protocols deposited at the clinical trials (Table 2); this suggestion was accepted in some reviews [42, 43] and recently included in the guidelines of the Association of Breast Surgery and the British Association of Plastic, Reconstructive, and Aesthetic Surgeons [44]. This document recognized that the RXT negatively affected the outcome but suggested that the use of ADM could potentially reduce the severity of capsular contracture.

In addition to capsular contracture, infection and implant failure seemed to occur more frequently in irradiated breasts [33]. Data we analysed here confirm this trend for all types of complications and reached the statistical significance in the case of skin necrosis and capsular contracture. The most severe outcome, implant failure, was also significantly enhanced, independently from the causes.

Several biases occur in the analysis. The influence of chemotherapy, in relation with the concomitant need for radiation therapy, was sporadically reported in details in the reviewed papers, as were other recognized risk factors, such as obesity, diabetes, smoking, and breast size [45–47]. The data retrieved from the analysis of the literature did not permit to stratify the observations in relation to the RXT; it is specified by some authors that the risk factors were evenly distributed among groups. Another source of perplexity could rise from the consideration that, for its relatively elevated cost [48], the use of ADM could be addressed to patients with a lower risk for complications, or with a more favourable stage of the cancer. This factor, poorly investigated until now [2, 38], could put the suggested protective effect under a more relative point of view.

## 5. Conclusions

Radiotherapy is generally considered a concomitant factor negatively influencing the dynamics of breast reconstruction, even when ADM is added. This fact, claimed by many studies published on this argument, seems to emerge from the meta-analysis presented here. The real impact of RXT on the success of breast reconstruction techniques must be better defined by studies falling in the level of evidence I or II, namely, protocols specifically designed to define the importance of the radiotherapy and planned as randomized, single-, or multicenter studies of adequate quality. Better assessment of anthropometric and behavioural conditions, morbidity, and stage of the cancer in the two groups, with or without RXT, should be added for clarity.

A better definition of histological evolution of the ADM after surgery in sites that were previously irradiated should also be of interest and even more in those undergoing RXT after the surgery, particularly in terms of formation of oxygen reactive species and radicals and collagen integrity.

## Figures and Tables

**Table 1:** Inclusion and exclusion criteria for quantitative meta-analysis. Studies marked with asterisk were considered for qualitative analysis.

Inclusion criteria	
Primary data from prospective and retrospective observational studies	
Human studies	
Studies that include data on prophylactic or therapeutic mastectomy for cancer	
Studies that stratify results on the basis of delivery of radiotherapy before or after initiation of reconstruction	
Studies based on single- or two-stage implant breast reconstruction	

Exclusion criteria	

Experimental studies performed in laboratory animals or “in vitro”	
Review, surgical technique description, or case report; studies with no relevant extractable outcomes*	
Studies focused solely on the elderly (>65 years)	
Studies not published in English	
Papers published before year 2005	

**Table 2:** Studies registered at the clinical trials registry (http://www.clinicaltrials.gov/) of the United States of America and at the international clinical trials registry platform (World Health Organisation, http://apps.who.int/trialsearch/) concerning the outcome of ADM-assisted breast reconstruction after mastectomy.

NCT	Status	Location	Expected NR	Principal aim	Radiotherapy
NCT 00616824	R	USA	60	D versus B	Exclusion criteria

NCT 00639106	NR	USA	98	A versus B	Exclusion criteria

NCT 00692692	NR	USA	36	E versus D	Not named

**NCT 00872859**	**NR**	**USA**	**196**	**A or D with/without RXT**	**Principal condition**

NCT 00956384	R	Canada	144	C versus E	Exclusion criteria

NCT 01027637	ND	USA	30	Defining the stretch parameters of A	Exclusion criteria

NCT 01222390	NR	USA	30	F versus E	Not named

NCT 01310075	R	USA	398	A versus SM	Exclusion criteria

NCT 01372917*	NR	USA	39	AM	Exclusion criteria; sterilization with *γ*-rays

NCT 01561287	R	USA	40	A, neovascularisation	Not named

NCT 01664091	NR	USA	32	ADM versus others, with/without RXT	**Principal condition, postsurgery**

NCT 01679223	NR	USA	60	AM, incorporation	Not named

NCT 1781299	R	UK	50	A RTU versus SM	Anamnestic record

NCT 01823107	R	USA	25	G	Exclusion criteria

NCT 01853436	R	Italy	60	S	Exclusion criteria

**NCT 01959867**	**NR**	**USA**		**SM versus B**	**Principal condition, prior to surgery**

ISRCTN 67956295	NR	UK	40	S versus SM	Not named

A: alloderm; ADM: acellular dermal matrix; AM: allomax; B: traditional reconstruction; C: 1-stage dermal matrix/implant procedure; D: DermaMatrix; E: 2-stage tissue expander/implant procedure; F: ContourProfile^©^ expander; G: Meso BioMatrix Acellular Peritoneum Matrix; ISCTNR: international standard randomised controlled trial number register; NCT: national clinical trial accession number; R: recruiting; NR: not recruiting; RTU: ready to use; RXT: radiation therapy; S: Strattice; SM: Surgimed; UK: United Kingdom; USA: United States of America. *Published results [4].

**Table 3:** List of the published clinical trials in which different types of acellular dermis matrices (ADMs) were used for the implant breast reconstruction. These papers, included in the meta-analysis, analytically reported the rate of complications in patients treated or not with radiotherapy (RXT). All studies but two (Nicolau et al., 2012, Canada, [7], and Potter et al., 2013, UK, [8]) were from groups operating in the USA or cooperating with American institutions. NR: not reported.

Reference	ADM	Period, location	Nr. Of Pts./Reconstr.	Follow-up
Gamboa-Bobadilla, 2006 [9]	Alloderm	2003-2004, Medical College Georgia	11/13	14 months

Bindingnavele et al., 2007 [10]	Alloderm	2004-2005, Univ. South California	41/65	10 months

Nahabedian, 2009 [11]	Alloderm	1997–2008, Georgetown Univ.	76/100	NR

Namnoum, 2009 [12]	Alloderm	NR, Atlanta Plastic Surg.	20/29	21 months

Colwell et al., 2011 [13]	Alloderm versus no ADM	2006–2010, Univ. Massachusetts	211/331 and NR/128	NR

Israeli and Feingold, 2011 [14]	Strattice versus Alloderm	2005–2009, Hofstra Univ.	44/77 versus 72/122	12–22 months

Joanna Nguyen et al., 2011 [15]	Alloderm versus no ADM	1998–2008, Harvard Med. School	NR/75 and 246	NR

Rawlani et al., 2011 [16]	Flex-HD	NR, Northwestern Univ.	84/121	44 weeks

Nicolau et al., 2012 [7]	Alloderm	2008–2010, McGill University Health Centre, Canada	46/73	

Salzberg et al., 2011 [17]	Alloderm	2001–2010, NY Med. College	260/466	29 months

Salzberg et al., 2013 [18]	Strattice	2008-2009, NY Med. College	54/105	41 months

Spear et al., 2012 [19]	Alloderm	2004–2010, Georgetown Univ.	289/428	10–14 weeks

Seth et al., 2012 [20]	Alloderm/Flex-HD versus no ADM	2006–2008, Northwestern Univ.	NR/393 and 199	23-24 months

Parks et al., 2012 [21]	Alloderm versus no ADM	2001–2011, private practice, USA	232/346 and 114/165	NR

Cheng et al., 2013 [22]	Alloderm	2008–2012, Univ. of Texas; Emory University and Mayo Clinic.	11/16	9 months

Clemens and Kronowitz, 2012 [23]	Human, porcine, and bovine	2008–2012, Univ. of Texas	364/548	NR

Hanna et al., 2013 [24]	Alloderm versus no ADM	2007–2010, Virginia Univ.	31/38 and 44/62	8–10 months

Patel et al., 2013 [25]	ADM (not specified) versus no ADM	2005–2012, Univ. of California	NR/74 and NR/118	34 and 38 months

Potter et al., 2013 [8]	Protexa	2011-2012, University of Bristol, UK	31/46	3 months

Weichman et al., 2013 [26]	Alloderm	2006–2011, NY University	23/46	19 months

**Table 4:** Statistics under a fixed model for each study included in the meta-analysis.

Study name	Odds ratio	Lower limit	Upper limit	*Z* value	*P* value	Relative weight	Std. residual
Gamboa-Bobadilla, 2006 [9]	0.486	0.018	12.929	−0.431	0.667	0.55	−1.09

Bindingnavele et al. 2007 [10]	2.750	0.256	29.561	0.835	0.404	1.06	−0.08

Nahabedian, 2009 [11]	4.030	1.336	12.159	2.473	1.338	4.89	0.53

Namnoum, 2009 [12]	2.429	0.082	72.046	0.513	0.608	0.52	−0.12

Colwell et al. 2011 [13]	2.654	1.327	5.304	2.761	0.006	12.44	−0.38

Israeli and Feingold, 2011 [14]	1.268	0.474	3.391	0.474	0.636	6.17	−1.78

Joanna Nguyen et al. 2011 [15]	0.253	0.029	2.221	−1.240	0.215	1.27	−2.25

Rawlani et al. 2011 [16]	2.803	1.013	7.757	1.985	0.047	5.76	−0.14

Nicolau et al. 2012 [7]	0.342	0.018	6.468	−0.716	0.474	0.69	−1.46

Salzberg et al. 2011 [17]	2.890	0.791	10.555	1.605	0.108	3.56	−0.06

Salzberg et al. 2013 [18]	0.733	0.038	14.044	−0.206	0.837	0.68	−0.94

Spear et al. 2012 [19]	9.114	5.778	14.377	9.503	0.000	28.74	5.64

Seth et al. 2012 [20]	1.896	0.878	4.094	1.628	0.103	10.07	−1.24

Parks et al. 2012 [21]	0.426	0.191	0.948	−2.091	0.036	9.32	−5.03

Cheng et al. 2013 [22]	29.000	0.780	1077.623	1.826	0.068	0.46	1.23

Clemens and Kronowitz, 2012 [23]	4.126	1.929	8.822	3.655	0.000	10.33	0.86

Hanna et al. 2013 [24]	0.217	0.022	2.131	−1.311	0.190	1.14	−2.27

Patel et al. 2013 [25]	0.231	0.009	6.107	−1.877	0.380	0.56	−1.54

Potter et al. 2013 [8]	36.000	3.453	375.309	2.996	0.003	1.09	2.09

Weichman et al. 2013 [26]	21.364	1.139	400.534	2.047	0.041	0.69	1.31

**Model: fixed**	**3.010**	**2.358**	**3.844**	**8.841**	**0.000**		

**Table 5:** Statistical evaluation of the results of meta-analysis. The test of null was performed for both the fixed and random models and was significant. The heterogeneity and Tau-squared tests applied to the fixed model are also shown.

Model	Test of null (2-tail)		Heterogeneity				Tau-squared			
	*Z* value	*P* value	*Q* value	df (*Q*)	*P* value	*I*-squared	Tau-squared	SE	Variance	Tau
Fixed	−8.841	0.000	75.243	19	0.000	74.748	1.015	0.603	0.364	1.008

**Table 6:** Rate of complications by group (no RXT versus RXT).

Complication	Number of studies	No RXT (%)	ES	RXT (%)	ES	*P* value
Overall	11	15.63	3.59	24.71	5.67	0.000
Infection	10	8.19	2.73	12.04	4.01	0.400
Skin necrosis	10	4.04	1.35	15.50	1.05	0.000
Seroma	9	3.61	1.20	4.60	1.53	0.045
Capsular contracture	6	2.88	1.09	11.90	4.50	0.033
Others	10	4.02	1.34	8.18	2.73	0.000
Failure	11	4.06	1.23	14.05	4.23	0.000
